# Calcium-permeable AMPA receptors on AII amacrine cells mediate sustained signaling in the On-pathway of the primate retina

**DOI:** 10.1016/j.celrep.2022.111484

**Published:** 2022-10-11

**Authors:** Kumiko A. Percival, Jacqueline Gayet, Roupen Khanjian, W. Rowland Taylor, Teresa Puthussery

**Affiliations:** 1 Herbert Wertheim School of Optometry & Vision Science, University of California, Berkeley, Berkeley, CA 94720-2020, USA; 2 Helen Wills Neuroscience Institute, University of California, Berkeley, Berkeley, CA 94720-2020, USA; 3 Casey Eye Institute, Oregon Health & Science University, Portland, OR 97239, USA; 4 Lead contact

## Abstract

Midget and parasol ganglion cells (GCs) represent the major output channels from the primate eye to the brain. On-type midget and parasol GCs exhibit a higher background spike rate and thus can respond more linearly to contrast changes than their Off-type counterparts. Here, we show that a calcium-permeable AMPA receptor (CP-AMPAR) antagonist blocks background spiking and sustained light-evoked firing in On-type GCs while preserving transient light responses. These effects are selective for On-GCs and are occluded by a gap-junction blocker suggesting involvement of AII amacrine cells (AII-ACs). Direct recordings from AII-ACs, cobalt uptake experiments, and analyses of transcriptomic data confirm that CP-AMPARs are expressed by primate AII-ACs. Overall, our data demonstrate that under some background light levels, CP-AMPARs at the rod bipolar to AII-AC synapse drive sustained signaling in On-type GCs and thus contribute to the more linear contrast signaling of the primate On- versus Off-pathway.

## In brief

Percival et al. describe how a night vision circuit contributes to functional asymmetries in signaling of the On- and Off-type midget and parasol ganglion cells, the major output neurons of the primate retina.

## Graphical Abstract

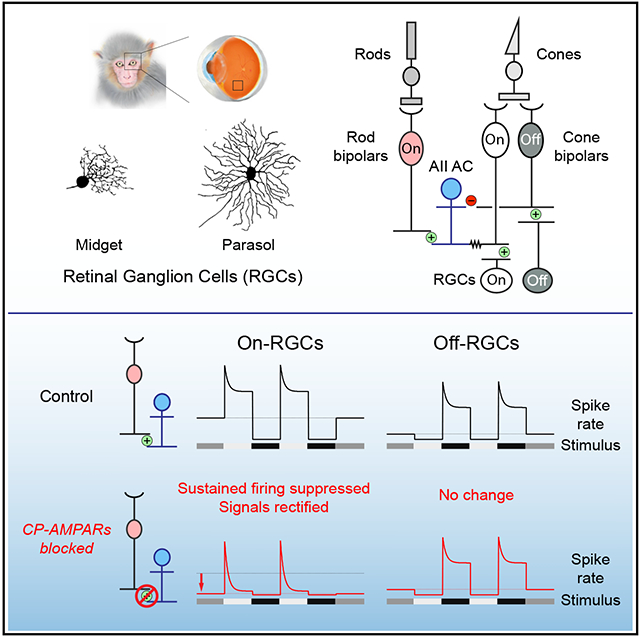

## INTRODUCTION

Sensory systems divide incoming information into parallel pathways, effectively mitigating losses associated with the limited signal-to-noise ratios of neural transmission. The two major parallel pathways in the visual system, the On and Off pathways, have opposing responses to luminance contrast changes. On-pathway cells are excited by positive contrast, whereas Off-pathway cells are excited by negative contrast. Although the two pathways receive a common input from the photoreceptors, the On and Off signals reaching the ganglion cells (GCs) are not simply mirror-symmetric with respect to contrast. Signals in On-type GCs are more linear, due to higher baseline firing that allows the GCs to modulate their firing rate around the mean level. Signals in Off-type GCs are less linear (more rectified) due to lower baseline firing rates that limit their capacity to respond to contrast increments (Chichilnisky and Kalmar, 2002; Cleland et al., 1973; Kaplan et al., 1987; Liang and Freed, 2010; Soto et al., 2020; Trong and Rieke, 2008; Turner and Rieke, 2016; Zaghloul et al., 2003). These differences in the dynamics of the On and Off pathways arise within the synaptic circuitry between the photoreceptor terminals and the inputs to GC dendrites. In primates, On/Off-pathway differences are seen in both the midget and parasol GCs, raising the possibility that a common circuit element might be involved (Chichilnisky and Kalmar, 2002; Soto et al., 2020; Turner and Rieke, 2016).

**Figure 1.**
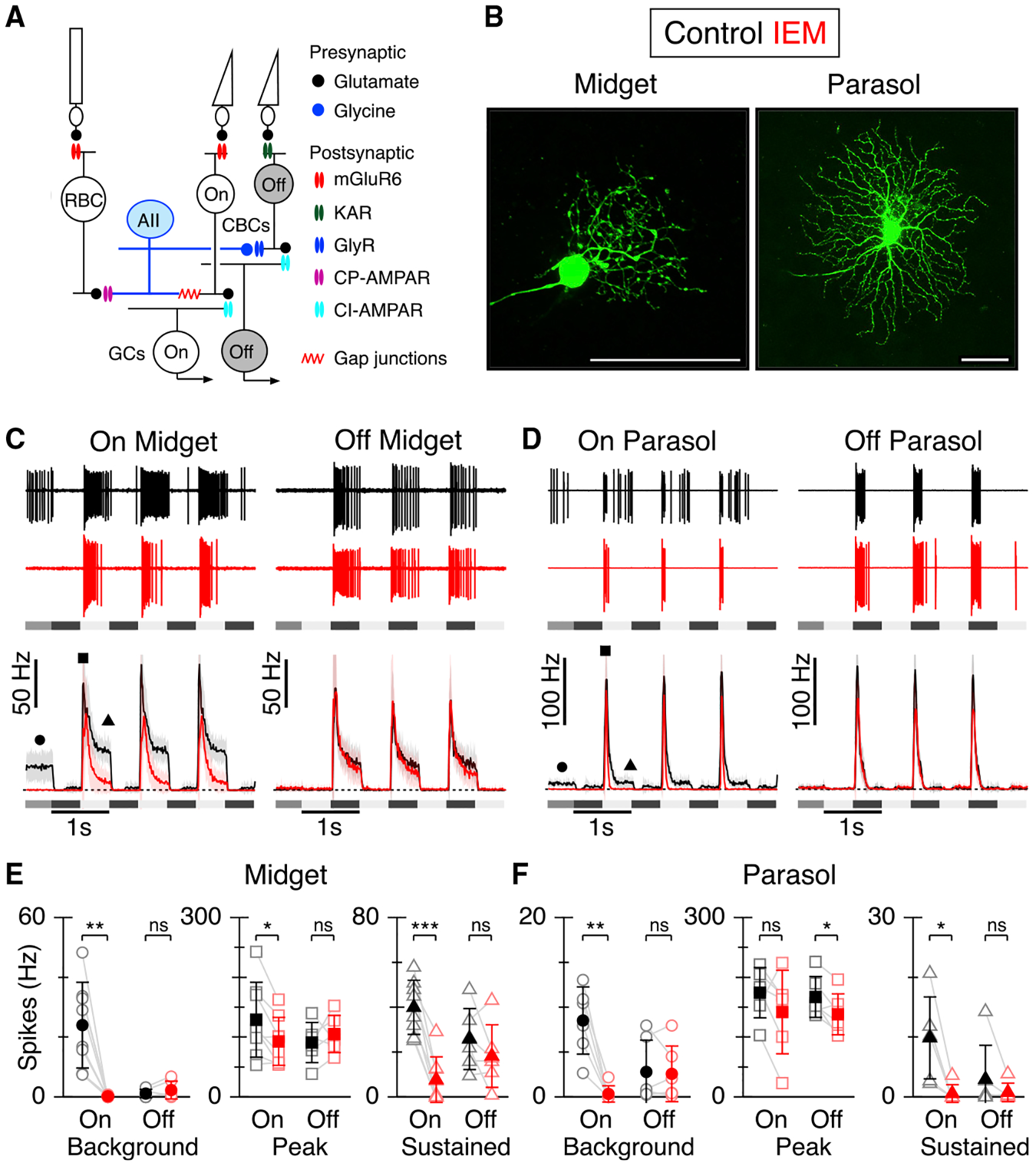
CP-AMPARs drive sustained firing in primate On-GCs (A) Schematic diagram showing the organization of the rod and cone signaling pathways and known location of different chemical and electrical synapses. (B) Dye fills showing examples of On-midget (left) and On-parasol (right) GC morphology. Scale bars, 50 μm. (C and D) Representative extracellular spike records from On- and Off-type midget (C) and parasol (D) GCs in control (black) and during application of IEM 1460 (50 μM, red). Peristimulus spike-time histograms (PSTHs) are shown beneath, with the stimulus timing and contrast indicated. The stimulus was a centered spot approximately the size of the excitatory receptive field, square-wave modulated at 80% contrast. The shading on the PSTHs shows ± 1 SD for the average responses from eight On-midget GCs, six Off-midget, six On-parasol GCs, and six Off-parasol GCs. (E and F) Spike rates measured from midget (E) and parasol (F) PSTHs at the time points indicated by the symbols in (C) and (D). Open symbols show individual cells, solid symbols show average ± 1 SD. *p < 0.05, **p < 0.01, ***p < 0.001.

Here, we tested whether the enhanced linearity of the On-pathway is due to activity of the AII amacrine cells (AII-ACs), which are key relay neurons in the primary rod pathway. Under steady scotopic and mesopic background light levels, AII-ACs receive tonic excitatory drive from rod bipolar cells (RBCs), and signal linearly by modulating their voltage around a mean level (Ke et al., 2014). This arrangement allows the AII-ACs to route signals into the On and Off pathways (Ke et al., 2014; Muller et al., 1988) via the cone bipolar cells, which in turn make inputs to the Off- and On-GCs (Figure 1A). The AII-ACs are thus well-positioned to provide a common linear input signal to On-midget and On-parasol GCs.

A conserved feature of mammalian RBC to AII-AC synapses is the presence of calcium-permeable AMPA receptors (CP-AMPARs; Ghosh et al., 2001; Kim and von Gersdorff, 2016; Mørkve et al., 2002; Pourcho et al., 2002; Singer and Diamond, 2003). CP-AMPARs are distinct from other AMPARs in that they either lack or contain an unedited, GluA2 subunit (Cull-Candy and Farrant, 2021; Hollmann et al., 1991). These receptors exhibit high calcium permeability, high single channel conductance (Swanson et al., 1997), and can be selectively blocked by the adamantane derivative, IEM-1460 (Magazanik et al., 1997). In this study, we exploit this pharmacological selectivity to test whether AII-ACs contribute to linear signaling in midget and parasol GCs, even at background levels under which rods might be expected to saturate. We show that CP-AMPARs are essential to support linear signaling in On-type midget and parasol GCs and provide evidence supporting the AII-ACs as the likely site of action.

## RESULTS

We recorded from On- and Off-type midget and parasol GCs from macaque monkey retina. Midget GCs were distinguished from parasol GCs by their smaller soma size, and relatively sustained responses during square-wave flicker stimulation. Parasol GCs had the largest soma sizes and relatively transient light responses (Crook et al., 2014; Puthussery et al., 2013, 2014; Silveira et al., 2004). In some cases, cell type was verified by examining morphology from dye fills at the end of the recordings (~20% of recorded cells, Figure 1B).

### CP-AMPARs drive sustained spiking in On-midget and On-parasol retinal GCs

Glutamatergic transmission at the first synapse between photoreceptors and bipolar cells is mediated by mGluR6 receptors (On-pathway) and kainate receptors (Off-pathway), with no significant involvement of AMPARs (Puthussery et al., 2014; Vardi et al., 2000). Given the absence of AMPA receptors at the photoreceptor-to-bipolar cell synapses (Figure 1A), we could test the effect of CP-AMPARs on signaling in the inner plexiform layer using the selective CP-AMPAR antagonist, IEM-1460 (IEM, 50 μM).

Midget and parasol GCs were stimulated using a square-wave contrast-modulated spot of light (1 Hz, 80% contrast) centered on the receptive field and matched to the size of the receptive field center (Figures 1C–1F). Bath-application of IEM suppressed the pre-stimulus background firing in both On-midget (n = 8 cells) and On-parasol (n = 6 cells) GCs (Figures 1C–1F, On-midget: control 24.0 ± 14.4 Hz; IEM 0.3 ± 0.4 Hz; p = 0.0023. On-parasol: control 8.5 ± 3.8Hz; IEM 0.4 ± 0.9 Hz; p = 0.0026). The sustained spiking during the On-phase of the light stimulus was also suppressed (On-midget: control 40.1 ± 12.1 Hz; IEM 7.8 ± 10.2 Hz; p = 7.4 × 10^−5^, On-parasol: control 9.9 ± 6.8 Hz; IEM 0.6 ± 1.5 Hz; p = 0.011). The peak firing rate at the beginning of each stimulus cycle was suppressed for On-midget cells and unaffected for On-parasol cells (On-midget: control 129.5 ± 63 Hz; IEM 93 ± 40 Hz; p = 0.013. On-parasol: Ctrl 174.4 ± 42 Hz; IEM 142 ± 70 Hz; p = 0.21). Although parasol GC responses are characteristically more transient than midget GCs, they too became more transient during CP-AMPAR blockade.

Compared with On-type GCs, the corresponding Off-type GCs showed little background spiking under identical recording conditions and spiking was largely unaffected by CP-AMPAR blockade (Figures 1C–1F). These results indicate that CP-AMPARs contribute to sustained spiking in On-type midget and parasol GCs, but not to the corresponding Off-type GCs.

### Blocking NMDARs does not recapitulate the effects of CP-AMPAR block

**Figure 2.**
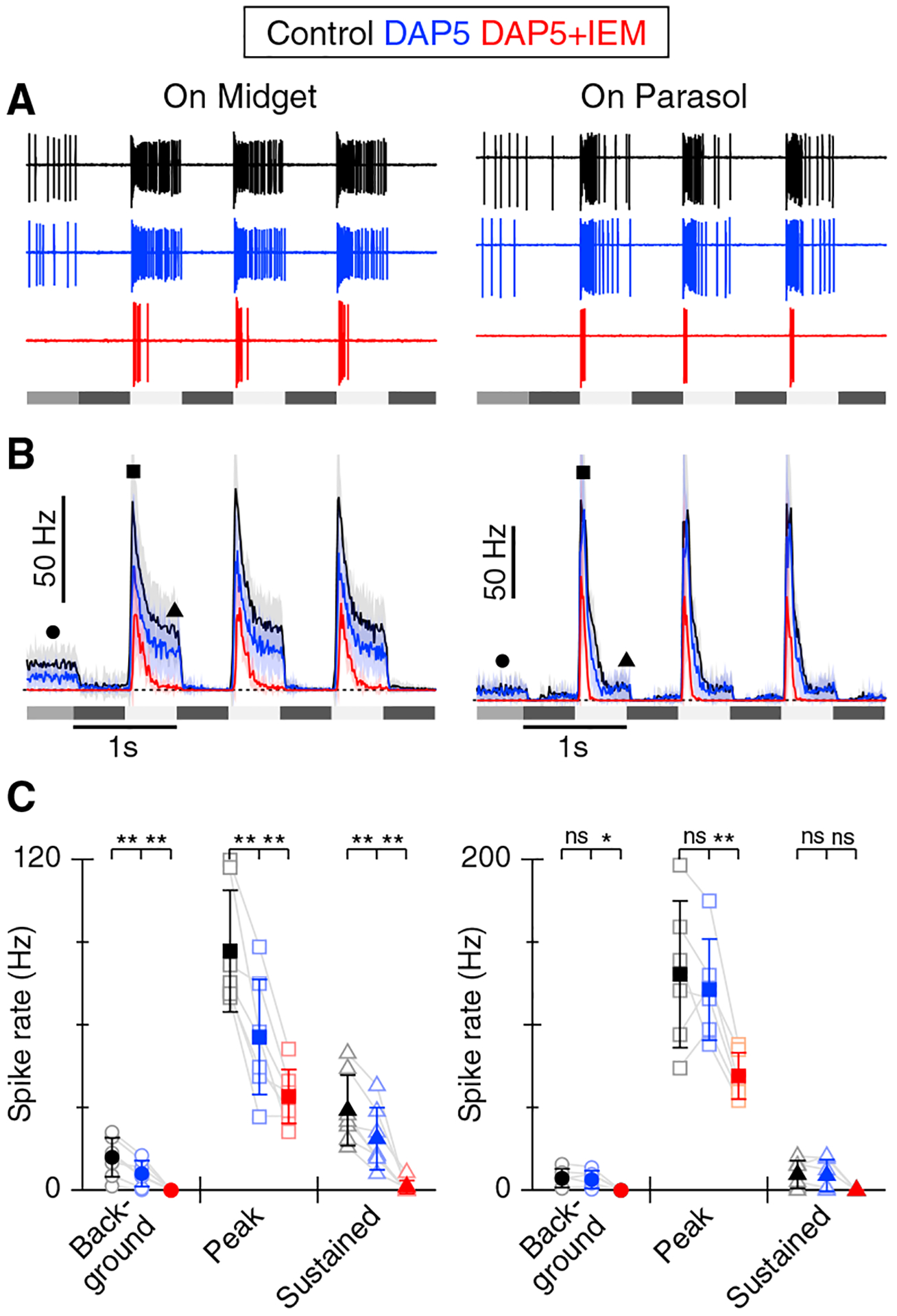
Blocking NMDA receptors does not selectively suppress sustained firing in On-GCs (A) Representative extracellular spike records in an On-midget and On-parasol GC in control (black), D-AP5 (blue), or in D-AP5 with subsequent addition of IEM1460 (red). The stimulus timing is shown beneath and the stimulus protocol is as in Figure 1. (B) Average PSTHs from seven On-midget and six On-parasol GCs with shading showing ± 1 SD. (C) Summary data showing background, peak, and sustained firing rate in On-midget (left) and On-parasol cells (right) as measured from PSTHs at the time points indicated by the symbols in (B). Error bars show ± 1 SD. *p < 0.05, **p < 0.01.

Primate GCs express AMPA and NMDA receptors (Cohen and Miller, 1994; Crook et al., 2014; Jacoby and Wu, 2001). NMDA receptors have slower gating kinetics and desensitize more slowly than AMPA receptors (Traynelis et al., 2010) and thus could contribute to sustained responses in GCs. Therefore, we tested whether sustained spike activity could be suppressed by blocking NMDARs with the competitive antagonist *D*-AP5 (50 μM). In On-midget GCs, blocking NMDARs reduced background spiking by 49% (p = 0.003), the peak light-evoked spiking by 36% (p = 0.0027), and the sustained component of light-evoked spiking by 36% (p = 0.0014; Figures 2A and 2B, n = 7 cells). Subsequent addition of the CP-AMPAR antagonist blocked the residual background spiking by 99% (control 11.9 ± 2.7 Hz, *D*-AP5 6.1 ± 1.8 Hz, D-AP5+IEM, 0.03 ± 0.05 Hz, p = 0.0015), the residual peak response by 38% (control 86.8 ± 8.3 Hz, *D*-AP5 55.5 ± 7.9 Hz, *D*-AP5+IEM, 34.0 ± 9.8 Hz, p = 0.0089), and the remaining sustained response by 94% (control 29.0 ± 4.9 Hz, D-AP5 18.7 ± 4.2 Hz, D-AP5+IEM, 1.2 ± 2.3 Hz, p = 0.0061, Figures 2A and 2B). In On-parasol GCs, blocking NMDARs had no effect on background firing or the peak or sustained light responses (p values 0.43, 0.50, and 0.68; Figures 2B and 2C, n = 6 cells). These results demonstrate that blocking NMDARs reduced overall spiking in On-midget GCs but did not have a disproportionate effect on the sustained response component. In the presence of the NMDAR antagonist, the CP-AMPAR blocker completely suppressed sustained spiking in On-midget and On-parasol GCs, but only partially suppressed the transient response component.

### Effects of blocking CP-AMPARs are independent of inhibitory circuits

The effects of CP-AMPAR blockade could be due to effects on inhibitory amacrine cell circuits (Diamond, 2011). For example, excitatory drive to some GCs in the mouse retina can be driven, at least in part, by direct disinhibition (Manookin et al., 2008; van Wyk et al., 2009). Although previous studies have not reported strong disinhibitory input to either On or Off-midget or parasol GCs (Crook et al., 2014), it was nonetheless important to determine whether the effects of CP-AMPAR blockade might be due to polysynaptic effects involving inhibitory neurotransmission.

**Figure 3.**
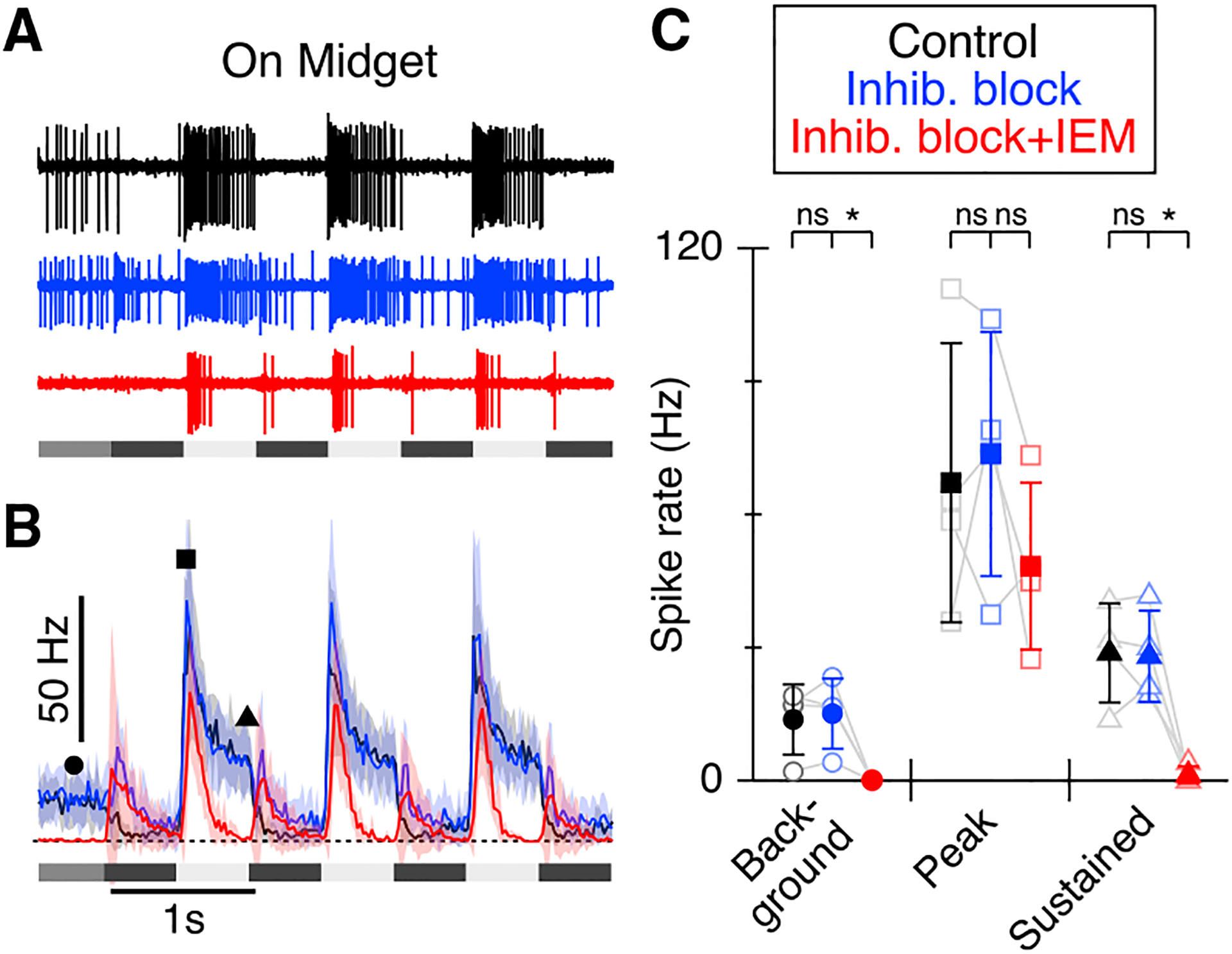
Inhibition has little effect on spiking responses in On-midget GCs (A) Representative extracellular spike records from an On-midget GC in control (black), with inhibition blocked (blue; GABA_A_, 10 μM SR95531, GABA_C_ 100 μM TPMPA, glycine, 1 μM strychnine), or in inhibitory blockers with subsequent addition of IEM1460 (red). Stimulus is as in Figure 1. (B) PSTHs averaged from four On-midget cells with shading showing ± 1 SD. (C) Summary data for measurements from PSTHs in (B) at the time points indicated by the symbols. Error bars show ± 1 SD. *p < 0.05.

We tested the effect of blocking inhibitory neurotransmission on the responses of the On-midget GCs. Simultaneous block of GABAergic (GABA_A_, 10 μM SR95531; GABA_C_, 100 μM TPMPA) and glycinergic inhibition (1 μM strychnine) had no discernible effect on the background spiking, peak light-evoked spiking, or sustained light-evoked spiking in On-midget GCs during the On-phase of the stimulus (p values 0.67, 0.52, and 0.84; Figure 3, n = 4 cells). Subsequent addition of the CP-AMPAR antagonist to the inhibitory cocktail had no significant effect on the peak response (p = 0.14) but suppressed the background spiking and the sustained responses as it did in the absence of inhibitory blockade (background spiking: control 13.8 ± 4.0 Hz; inhibitory blockers 15.1 ± 4.0 Hz; inhibitory blockers + IEM 0.1 ± 0.1 Hz; p = 0.033. Sustained spiking: control 28.8 ± 5.6 Hz; inhibitory blockers 28.0 ± 5.1 Hz; inhibitory blockers + IEM 1.4 ± 1.0 Hz; p = 0.017). Blocking inhibition unmasked a small excitatory Off response in On-midget GCs that was not evident under control conditions (Crook et al., 2014) and was unaffected by the CP-AMPAR antagonist. This component was not evident during IEM application in the absence of inhibitory blockers (cf Figures 1 and 2), indicating that the inhibitory ACs that normally suppress the Off-excitation do not rely on CP-AMPARs for activation. Overall, these results demonstrate that the effects of blocking CP-AMPARs cannot be explained simply by indirect, polysynaptic modulation of inhibitory circuits.

### CP-AMPARs drive a tonic excitatory input to On-GCs

**Figure 4.**
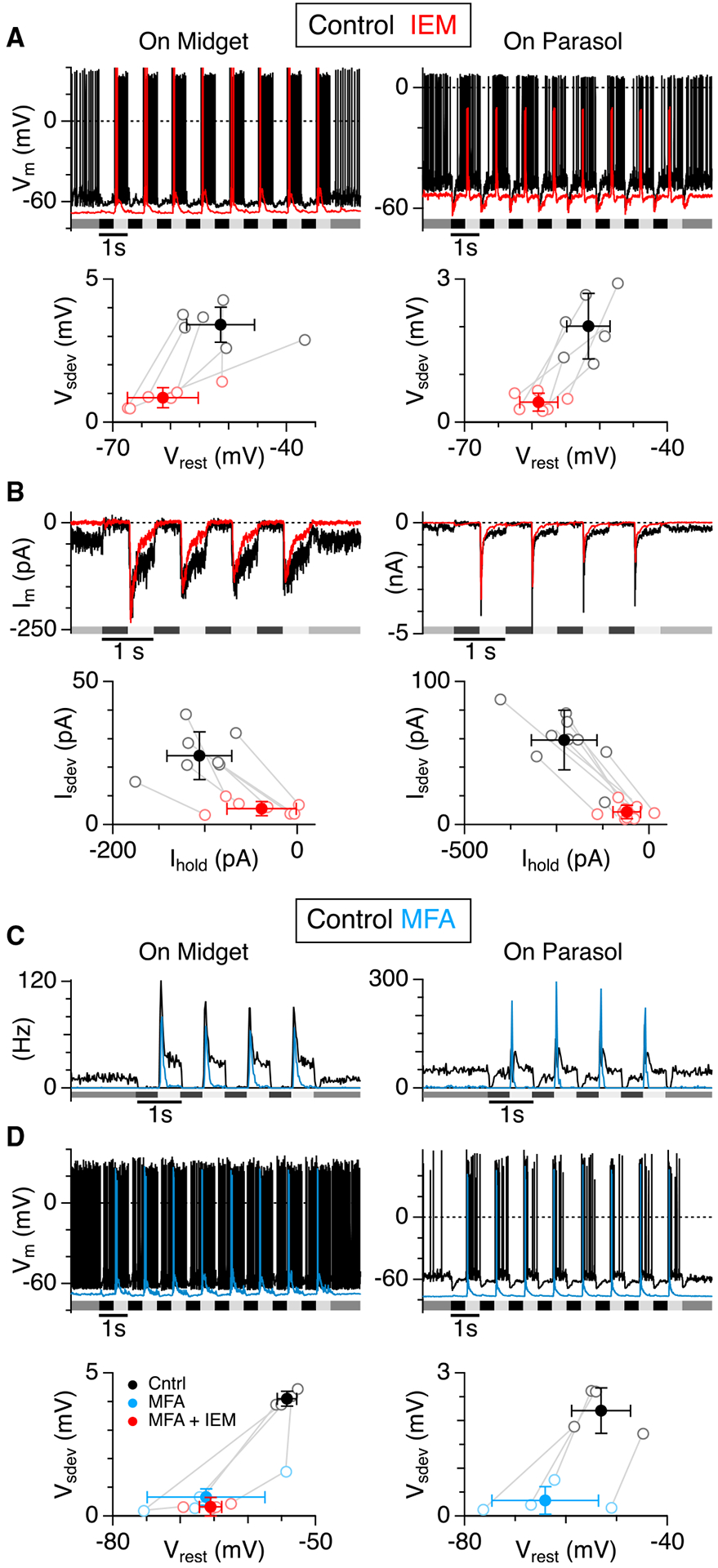
Sustained excitation of On-type midget and parasol cells is suppressed by a CP-AMPAR antagonist and a gap-junction blocker (A) Top panels: Examples of voltage recordings from an On-midget and an On-parasol GC in control (black) and in the presence of IEM (50 μM, red). Timing for the light stimulus, a 200-μm-diameter spot centered on the receptive field, is shown beneath the traces. Lower panels show the average resting voltage noise (V_sdev_) plotted against membrane potential (V_rest_) for six On-midget and six On-parasol GCs before and after IEM. The solid symbols with error bars show the means ± 1 SD. (B) Same format as for (A) showing membrane currents recorded in an On-midget and an On-parasol GC. Lower panels show the standard deviation of the current-noise plotted against the holding current at −60 mV for seven On-midget and nine On-parasol GCs before and after IEM. (C) PSTHs generated from 20 trials in two On-midget and one On-parasol cell in control (black) and after application of meclofenamic acid (MFA, 100 μM, blue). (D) Same format as for (A) showing voltage recordings from example On-midget and On-parasol GCs. Lower panels show summary data for four On-midget and four On-parasol GCs before and during application of MFA (blue). The red symbols show the lack of additional effect upon addition of IEM in the On-midget cells.

We next tested whether the CP-AMPAR antagonist blocked sustained excitatory input to the GCs. To this end, we measured the effect of CP-AMPAR blockade on the voltage and current responses in GCs during whole-cell patch-clamp recordings. If the GCs are tonically depolarized by the summation of EPSCs from convergent synaptic inputs, hyperpolarization should be accompanied by a decrease in the standard deviation of the baseline voltage signal. Application of the CP-AMPAR antagonist hyperpolarized On-midget GCs by −10.0 ± 10.4 mV, but the effect did not reach significance (p = 0.065, n = 6); however, the antagonist caused a significant decrease in the standard deviation of the baseline voltage signal from 3.4 ± 0.61 mV to 0.86 ± 0.35 mV (p = 4.7 × 10^−5^, n = 6, Figure 4A). Similarly, the CP-AMPAR antagonist reduced sustained spiking in On-parasol GCs, hyperpolarized the cells by −7.4 ± 3.9 mV (p = 5.7 × 10^−3^, n = 6), and decreased the standard deviation of the baseline voltage signal from 2.0 ± 0.68 mV to 0.42 ± 0.19 mV (p = 1.9 × 10^−3^, n = 6, Figure 4A). The effects of IEM on the baseline voltage were mirrored in voltage-clamp experiments measuring membrane current. Application of IEM blocked a standing inward current and reduced the standard deviation of the baseline current (Figure 4B). At −60 mV, the CP-AMPAR antagonist suppressed the inward current by 70 ± 23 pA in On-midget GCs (p = 2.2 × 10^−4^, n = 7), and 170 ± 70 pA in On-parasol GCs (p = 9.1 × 10^−5^, n = 9). There was a corresponding reduction in the standard deviation of the baseline current from 24.0 ± 8.3 pA to 5.6 ± 2.4 pA (p = 2.3 × 10^−4^) for the On-midget GCs, and from 59.1 ± 20.7 pA to 8.9 ± 4.8 pA (p = 2.3 × 10^−5^) for the On-parasol GCs. It is evident that the tonic inward current was modulated by light as it was suppressed during the Off-phase of the light stimulus (Figure 4B). In On-midget GCs, the Off-phase of the light stimulus suppressed 31.5 ± 19.5 pA of baseline inward current, whereas the average suppression was only 3.7 ± 4.9 pA in the presence of IEM (p = 4.5 × 10^−3^). Similarly, in the On-parasol GCs, the Off-phase of the light stimulus suppressed 85.0 ± 47.8 pA of baseline inward current in control conditions, but only 6.6 ± 15.2 pA in the presence of IEM (p = 1.2 × 10^−3^). These results support the hypothesis that a tonic inward current depolarizes both midget and parasol GCs and drives sustained spiking. In the experiments that follow, we sought to determine the locus of the CP-AMPARs within the circuit.

### A gap-junction blocker mimics and occludes the effects of the CP-AMPAR antagonist

CP-AMPARs are expressed at synapses between RBCs and AII-ACs (Mørkve et al., 2002; Osswald et al., 2007; Singer and Diamond, 2003). AII-ACs, in turn, make excitatory gap-junction connections with On-CBCs (Figure 1A) (Chun et al., 1993; Kolb and Famiglietti, 1974; Veruki and Hartveit, 2009). Tonic activation of CP-AMPARs on AII-ACs is expected to depolarize the On-CBCs through the gap-junction connections, and thus generate the sustained transmitter release that drives the sustained signals in On-GCs. We tested this hypothesis by applying the gap-junction blocker, meclofenamic acid (MFA, 100 μM), to block the connection between the AII-ACs and the On-CBCs (Veruki and Hartveit, 2009). As expected, MFA reduced background spiking and the sustained component of the light responses in On-midget and On-parasol GCs (Figure 4C, n = 2 On-midget, n = 1 On-parasol). Voltage-recording from On-type midget and parasol GCs showed that MFA had a similar effect to the CP-AMPAR blocker (Figure 4D). MFA hyperpolarized On-midget GCs by an average of −11.9 ± 8.1 mV (n = 4) but the effect did not reach significance (p = 0.061); however, there was a concomitant, significant decrease in the standard deviation of the membrane potential from 4.1 ± 0.27 mV to 0.67 ± 0.62 mV (p = 0.0010, n = 4). Similarly, MFA hyperpolarized On-parasol GCs by −11.1 ± 5.4 mV (p = 0.026, n = 4) and decreased the standard deviation of the membrane potential from 2.2 ± 0.48 mV to 0.32 ± 0.29 mV (p = 0.0017, n = 4, Figure 4D). If MFA and IEM both block transmission from RBCs through AII-ACs to On-type CBCs, then prior application of MFA should occlude any effect of subsequent co-application of IEM. We tested this prediction in the sample of On-midget GCs by adding 50 μM IEM to the bath solution after reaching steady-state with MFA. The addition of IEM had no further effect on the voltage variance or the resting membrane potential (Figure 4D, red). The similar effects of MFA and IEM suggest a role for the primary rod pathway, and specifically, the AII-ACs, in generating sustained spiking in On-GCs.

### CP-AMPARs mediate a tonic excitatory conductance with similar temporal properties in On-type midget and parasol GCs

**Figure 5.**
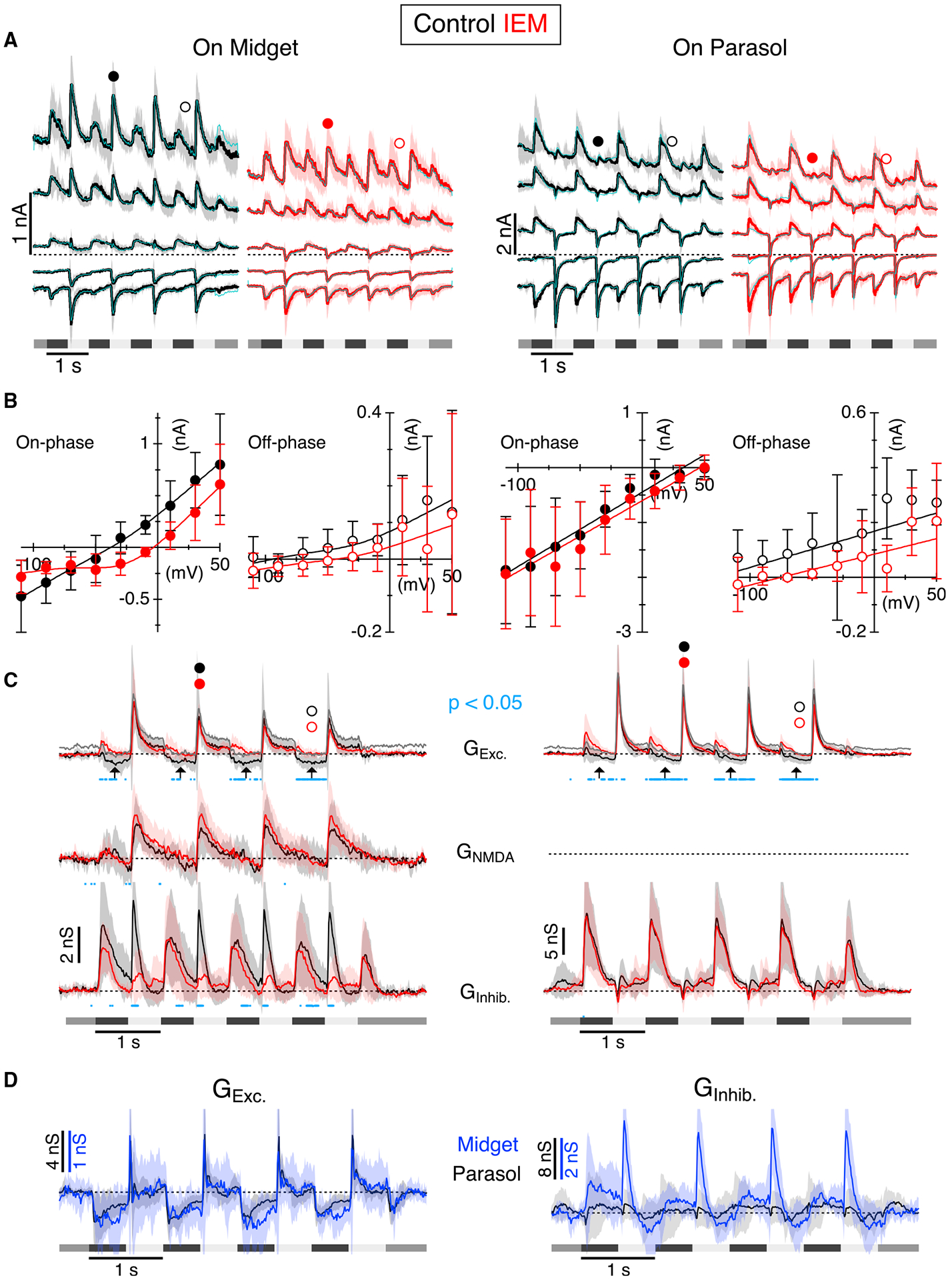
Blocking CP-AMPARs suppresses a sustained excitatory conductance in On-type midget and parasol GCs (A) Membrane currents averaged from six On-midget and six On-parasol GCs at a range of holding potentials during stimulation with a 200-μm-diameter centered spot flickered at 1 Hz. Stimulus timing is shown beneath the traces. The cyan overlays show the currents re-calculated from the conductances in (C). (B) Average current-voltage relations measured at the time points indicated by the corresponding symbols in (A). Solid lines show fits used to calculate conductances in (C). (C) Average light-evoked synaptic conductances calculated for the GCs. Blue dots indicate time points showing a significant difference (p < 0.05) in amplitude between control and IEM (paired t test). The apparently negative G_Exc_ during the Off-phase of the stimulus, highlighted by the arrows, is due to suppression of baseline (pre-stimulus) excitatory conductance. The gray traces in the top row show the predicted magnitudes of the excitatory conductances in control, assuming that the tonic excitation is completely suppressed during the Off-phase of the light stimulus. (D) Conductance blocked by the CP-AMPAR antagonist in the six midget and six parasol GCs, calculated by subtracting the respective conductances in the presence of IEM from those in control. Traces have been scaled to compare the time courses, as indicated by the vertical calibration bars. Shading and error bars show ±1 SD.

Signals from AII-ACs diverge to different On-CBC types and thus could represent a common source driving sustained visual responses in both midget and parasol GCs. A common source might be evident as a high degree of correlation between the signals in the On-GCs. To test this prediction, we compared the time course of the light-evoked excitatory and inhibitory synaptic conductances in On-midget and On-parasol GCs sampled in the same preparations under identical experimental conditions. We measured net light-evoked synaptic currents at a range of membrane potentials. Synaptic currents were evoked with a square-wave flickering spot (200 μm diameter, 1 Hz, 80% contrast, Figure 5A). The magnitudes of the light-evoked synaptic conductances were calculated from fits to the current-voltage (I-V) relations (Figures 5B and 5C). During the On-phase of the stimulus, On-midget GCs displayed nonlinear I-V relations (Figure 5B). This nonlinearity was particularly evident in the presence of the CP-AMPAR antagonist, due to the suppression of a transient inhibitory conductance, which presumably arose from an amacrine cell driven by CP-AMPARs. Such nonlinear I-V relations are consistent with reports of NMDAR-mediated synaptic input in On-midget GCs (Crook et al., 2011). The excitatory conductance in the On-parasol GCs was well fit by a linear I-V (Figure 5B), suggesting a smaller NMDAR-mediated contribution to On-parasol GCs, a finding in line with previous results (Crook et al., 2014).

In both On-GC types, the CP-AMPAR antagonist blocked a negative excitatory conductance during the Off-phase of the stimulus, consistent with suppression of a tonic, background excitatory input to these cells (Figure 5C, see also Figure 4B). The NMDAR component in the On-midget GCs was unaffected by the CP-AMPAR antagonist (G_NMDA_ Figure 5C), but transient inhibition at the onset of the On-phase of the stimulus was suppressed. The antagonist had no effect on the inhibition in On-parasol GCs (Figure 5C). The effects of the CP-AMPAR antagonist appear to be consistent with the effects on the spiking responses, namely, suppression of baseline activity and sustained responses with little effect on the transient response during a flickering stimulus.

To further test the notion that the AII-ACs are a common source driving the tonic excitation of both On-midget and On-parasol GCs, we compared the time course of the CP-AMPAR-sensitive responses in the two cell types. To do so, we calculated the net conductance suppressed by the CP-AMPAR antagonist by subtracting the conductance measured in the presence of IEM from the control conductance. In line with our prediction, after scaling by a factor of 4 to adjust for the magnitudes of the inputs, the components of the excitation sensitive to the CP-AMPAR antagonist displayed very similar time courses in the two cell types (Figure 5D). The corresponding inhibitory components were poorly correlated, since the antagonists had minimal effects on inhibition in the On-parasol GCs, but blocked transient On-phase inhibition in the On-midget GCs (Figure 5D). These results further support the notion that CP-AMPARs in AII-ACs provide a common source that drives sustained inputs to primate On-GCs.

### AII-ACs express CP-AMPARs in macaque retina

**Figure 6.**
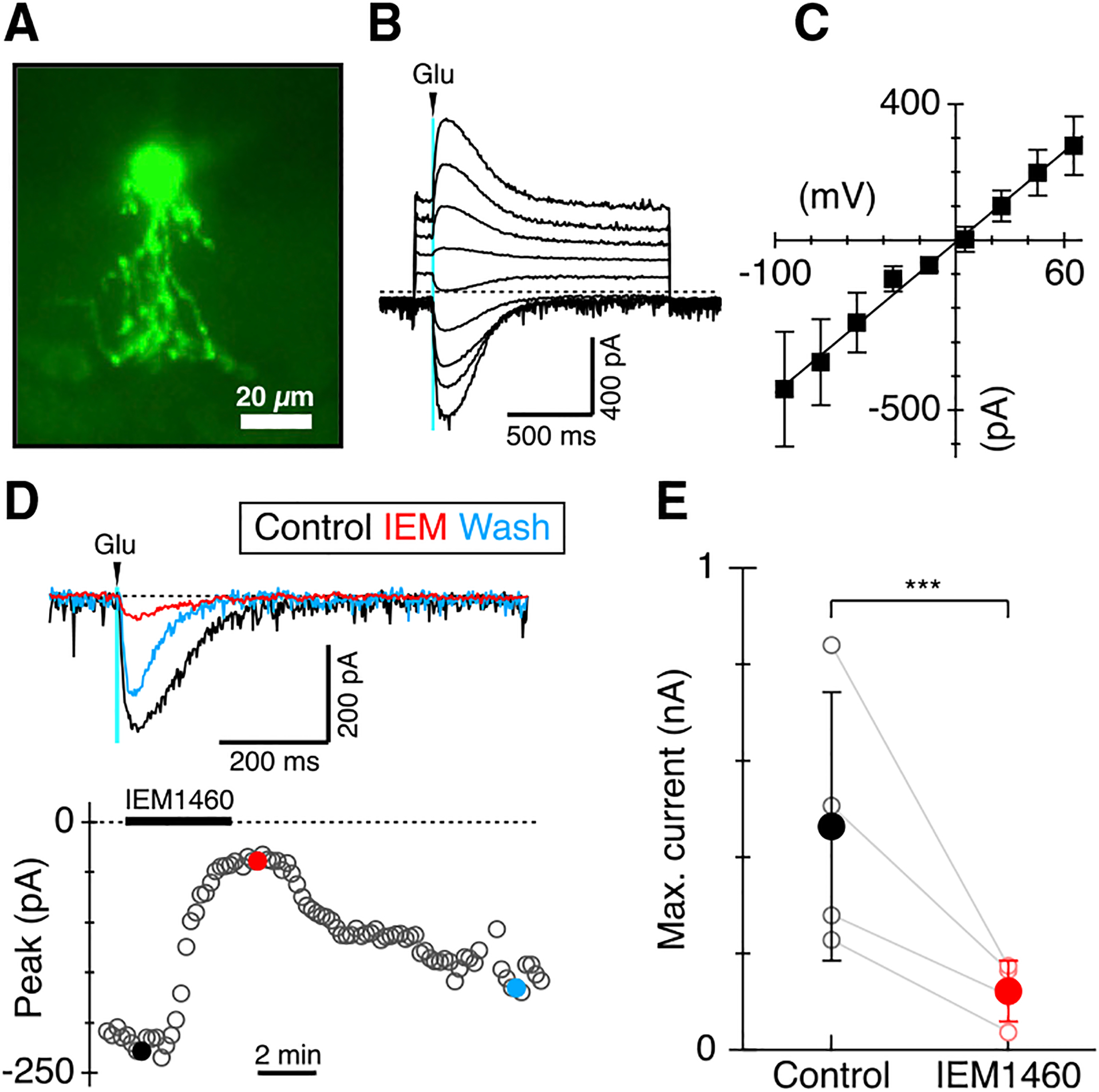
IEM suppresses glutamate-evoked currents in AII-ACs (A) Example of an AII-AC filled with Alexa 488 hydrazide during whole-cell recording in macaque retinal slices. Currents in AII-ACs were evoked by 20-ms puffs of L-Glu to the S5 region directly beneath the somas of the cells. Scale bar, 20 μm. (B) Currents in an AII-AC evoked by L-Glu puffs at holding potentials from −95 mV to +65 mV in 20-mV increments. (C) Average current-voltage relation for L-Glu evoked currents from seven AII-ACs. Solid line shows a linear regression to the average amplitudes, consistent with a conductance of 4.4 nS and reversal potential of +0.9 mV. Error bars show ± 1 SD. (D) L-Glu evoked currents in an example AII-AC (top panel) at timepoints before (black), during (red), and after partial washout (wash, blue) of 50 μM IEM-1460. Bottom panel shows the maximum amplitude of the inward L-Glu evoked current recorded at ~10-s intervals. The solid bar shows the timing of a 3.6-min application of IEM1460. Colored dots mark the timepoints for the example traces shown in the upper panel. (E) Summary data showing average suppression of L-Glu evoked currents in four AII-ACs. Solid symbols show the means with error bars showing ± 1 SD. ***p < 0.001.

The results above suggest that macaque AII-ACs express CP-AMPARs in line with earlier immunohistochemical studies (Ghosh et al., 2001). In order to obtain more direct evidence for CP-AMPAR expression, we measured currents elicited by focal application of L-glutamate (L-Glu, 0.5 mM) to AII-AC dendrites in slices of peripheral macaque retina (Figure 6). L-Glu was applied to stratum 5 of the inner plexiform layer IPL, where RBCs make synaptic input to AII-ACs. The L-Glu-evoked current-voltage relation was linear and reversed at the excitatory reversal potential (n = 7 cells, Figures 6B and 6C). The reversal potential indicates that glutamate activates pure excitation, with little if any indirect activation of inhibition. The linearity of the current-voltage relation, down to the most negative membrane potentials, indicates minimal activation of extrasynaptic NMDA receptors, which have been reported previously in rat AII-ACs (Kothmann et al., 2012). L-Glu activated a mix of AMPARs, with the majority being CP-AMPAR as evident from the effect of IEM (50 μM), which suppressed 73% ± 11% of the evoked current (Figures 6D and 6E, one-sample t test, p = 0.0009, n = 4). The antagonist effects were reversible in two cells that were held long enough to obtain partial washout (Figure 6D).

**Figure 7.**
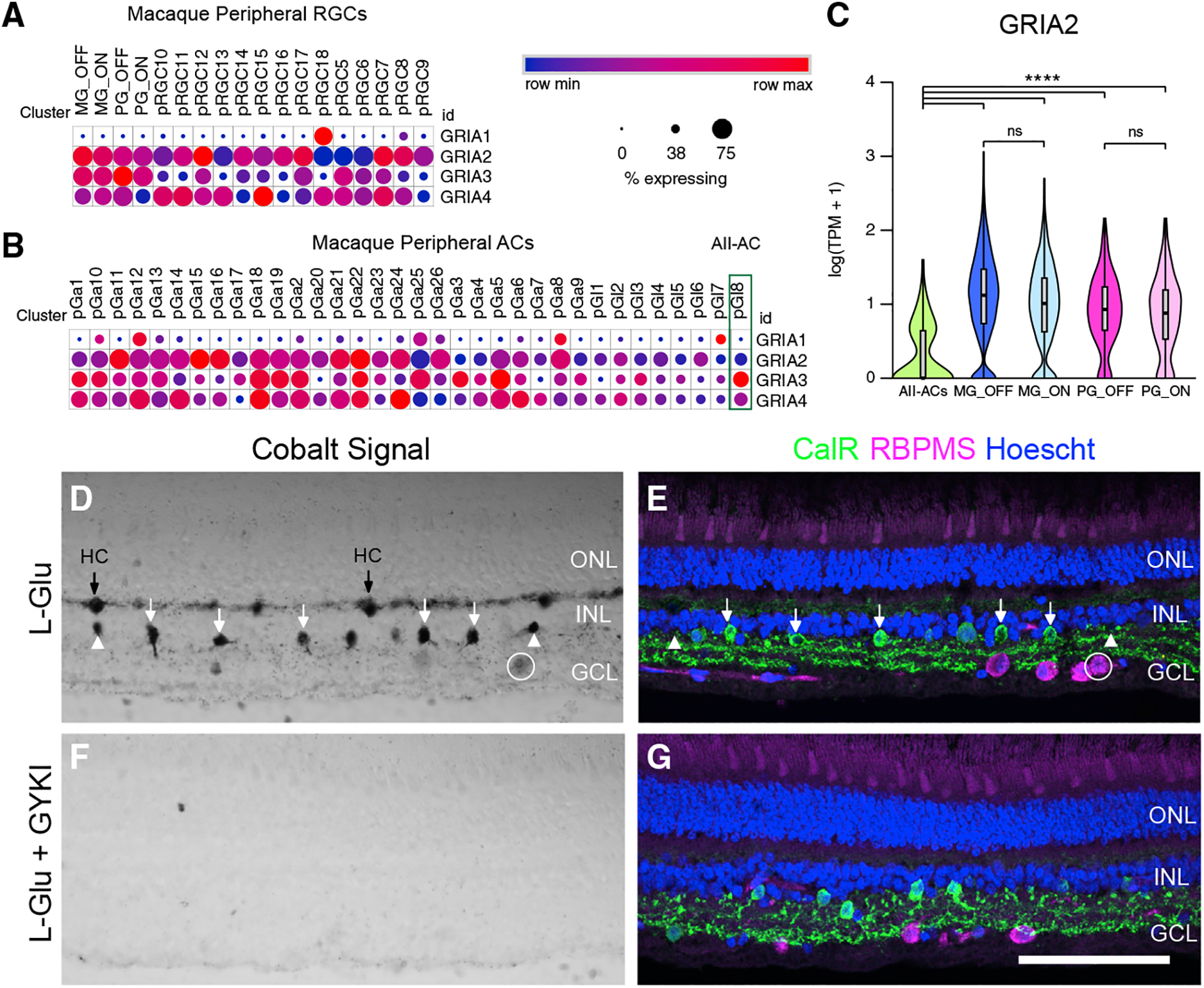
AII-ACs show lower GRIA2 expression and CP-AMPAR-mediated cobalt uptake than RGCs (A and B) Dot plots showing relative transcript expression of the AMPAR subunits, *GRIA1–4*, in macaque peripheral RGC (pRGC) clusters (A) and peripheral amacrine cells (B). MG, midget ganglion; PG, parasol ganglion; pGa, GABAergic amacrine cells; pGl, glycinergic amacrine cells. pGl8 is the AII-AC cluster (green rectangle in B). Circle size corresponds to the percentage of cells in the cluster expressing the gene, and intensity corresponds to the relative transcript count in expressing cells. Raw data from dataset of Peng et al. (2019). (C) Violin plot showing expression of *GRIA2* in AII-ACs and On- and Off-midget and parasol RGCs. Overlying boxplots show median/quartiles, whiskers show min/max values. ****log_2_ fold difference >2 and p < 0.00001. ns, <2-fold difference and/or p > 0.05. (D and F) Transmitted light images showing cobalt uptake signal with L-glutamate (D) or with L-glutamate in the presence of GYKI 53655 (80 μM) (F). (E and G) Same sections as in (D) and (F) immunolabeled with the pan-RGC marker, RBPMS, and calretinin (CalR), a marker of AII-ACs. Cell nuclei are labeled with Hoescht. HC, horizontal cells. White arrows show CalR+ amacrine cells that also show cobalt uptake. White arrowheads show examples of cobalt uptake in amacrine cells that are not labeled with CalR. An example of low-level cobalt uptake in an RGC in the GCL (white circle). Fluorescence images show maximal projections of four z-planes acquired near the tissue surface. Scale bar in (G) applies to (D–G), 100 μm. GCL, ganglion cell layer; INL, inner nuclear layer; ONL, outer nuclear layer.

The results above suggest that IEM suppresses RGC light responses through effects on AII-ACs; however, they do not discount the possibility that CP-AMPARs are also expressed on the RGCs themselves. To address this question, we analyzed transcript levels of the AMPAR subunits *GRIA1*, *GRIA2*, *GRIA3*, and *GRIA4* from an existing single-cell transcriptomic dataset from macaque retina (Peng et al., 2019; GEO: GSE118480). *GRIA2*, *GRIA3*, and *GRIA4* expression levels were relatively high in On-type and Off-type midget and parasol GCs relative to other RGC types (Figure 7A) suggesting high levels of calcium impermeable-AMPAR expression (i.e., receptors that contain *GRIA2*). By contrast, AII-ACs showed high levels of *GRIA3* and *GRIA4*, but lower levels of *GRIA2* compared with other amacrine cell types (Figure 7B), suggesting higher expression of CP-AMPARs. *GRIA2* levels were higher in midget and parasol RGCs compared with AII-ACs (log fold difference between means: MG OFF versus AII-AC 3.12, MG ON versus AII-AC 3.45, PG OFF versus AII-AC 2.91, PG ON versus AII-ACs 2.74, all comparisons significant to p < 0.0001 by t test, Figure 7C). *GRIA2* expression was higher in Off-midget versus On-midget RGCs (mean log(TPM+1), 1.07 ± 0.60 versus 0.97 ± 0.57, p < 0.0001), but the log fold difference in means was only 1.11. There was no significant difference in *GRIA2* expression between Off-parasol and On-parasol RGCs (mean log (TPM+1), 0.90 ± 0.49 versus 0.85 ± 0.53, p = 0.24, log fold difference 1.06). Taken together, these data are consistent with low levels of *GRIA2* expression (i.e., higher CP-AMPAR expression) in AII-ACs versus RGCs. Importantly, since *GRIA2* levels were similar in the On- and Off-type RGCs, the selective effects of IEM on the On-pathway cannot be explained by differential expression of CP-AMPARs at the level of the RGCs.

To further substantiate this conclusion, we used a functional cobalt staining method (Aurousseau et al., 2012; Osswald et al., 2007; Pourcho et al., 2002) to track CP-AMPAR activation in macaque retinal neurons (Figures 7D–7G). We stimulated cobalt uptake with 10 mM L-Glu as in prior studies (Aurousseau et al., 2012; Osswald et al., 2007; Pourcho et al., 2002) then immunolabeled retinas with cell-type-specific markers to classify the cell types showing uptake. We detected strong cobalt uptake in horizontal cells and a sparse subset of amacrine cells (Figure 7D). Double labeling with calretinin, a marker of primate AII-ACs (Kolb et al., 2002; Mills and Massey, 1999; Strettoi et al., 2018; Wässle et al., 1995), confirmed that the majority of cobalt-loaded cells in the inner nuclear layer were AII-ACs (Figure 7E), but some non-AII-ACs also showed cobalt uptake (Figure 7E), consistent with transcriptomic data showing low *GRIA2* levels in other cell types. Whereas strong cobalt loading was observed in the inner nuclear layer, the ganglion cell layer lacked similar loading, consistent with studies of normal rat retina (Cueva Vargas et al., 2015; Osswald et al., 2007). Occasional RGC somas showed a much weaker cobalt signal, an example of which is shown in Figure 7E. When glutamate was applied in the presence of 80 μM GYKI-53655, an antagonist that inhibits AMPAR channel gating, no cobalt uptake was detected, consistent with uptake through AMPARs and ruling out contributions of other cation-permeable channels such as NMDARs (Figures 7F and 7G). Similar experiments performed in mouse retina yielded similar results (n = 4, data not shown), and are consistent with findings in rat retina (Osswald et al., 2007). In summary, the data suggest high expression of CP-AMPARs in primate AII-ACs and HCs and negligible expression in RGCs. Together with the functional data, these results strongly suggest that CP-AMPAR antagonists suppress sustained responses in On-RGCs by blocking CP-AMPARs on AII-ACs.

## DISCUSSION

We have shown that CP-AMPARs drive sustained excitation to primate On-type midget and parasol GCs, while having little effect on the corresponding Off-type GCs. The effects of CP-AMPAR blockade persisted in the presence of inhibitory blockers and could be mimicked and occluded by gap-junction blockers. The time courses of the CP-AMPAR-sensitive inputs to On-midget and On-parasol GCs were similar, consistent with a common source (Figure 5D). Given the well-established circuitry connecting AII-ACs with GCs, we propose that a tonic excitatory drive from RBC→AII-ACs→On-CBCs→On-GCs generates background firing in On-type GCs that allows them to signal contrast more linearly. Below we discuss the evidence supporting this model.

### Site of action of CP-AMPAR antagonists

The observed effects of CP-AMPAR blockade are consistent with CP-AMPARs playing a major role in transmission at the RBC to AII-AC synapse (Jones et al., 2014; Mørkve et al., 2002; Singer and Diamond, 2003). The AII-ACs are critical interneurons in the primary rod pathway. They receive excitatory inputs from RBCs and route these signals to the On and Off pathways through sign-conserving gap-junction connections with On-CBCs, and sign-inverting glycinergic synapses with Off-CBCs (Bloomfield and Dacheux, 2001; Demb and Singer, 2012), (Figure 1A). We propose that blocking CP-AMPARs hyperpolarizes the AII-ACs, which in turn hyperpolarizes the On-CBCs. As the On-CBCs hyperpolarize below the threshold for glutamate release, their outputs become more rectified, and, in the absence of the input from the AII-ACs, are then driven entirely by direct synaptic inputs from cones. Several lines of evidence support this interpretation. First, after scaling for magnitude, we found that the time course of CP-AMPAR-sensitive currents in On-type midget and parasol GCs were remarkably similar (Figure 5D), consistent with the AII-AC providing a common input to both cell types. Second, the effects of the gap-junction blocker, MFA, were similar to that of the CP-AMPAR blocker, which is expected given that AII-ACs pass rod signals to On-CBCs via gap junctions (Kolb and Famiglietti, 1974; Veruki and Hartveit, 2002, 2009). Third, we show that glutamate-evoked currents in AII-ACs are strongly suppressed by CP-AMPAR blockade and that AII-ACs are one of only a few cell types that show strong cobalt uptake in the presence of glutamate. Finally, single-cell transcriptomic data are consistent with higher levels of CP-AMPAR expression in AII-ACs compared with On- and Off-GCs. Thus, the observed effects of CP-AMPAR blockade on the On-RGCs can be largely attributed to the selective expression of CP-AMPARs at the rod bipolar to AII-AC synapse.

Could CP-AMPARs at the On-CBC to On-GC synapse also contribute to the observed effects of IEM-1460? Prior studies have shown low levels of CP-AMPARs in rodent GCs (Cueva Vargas et al., 2015; Osswald et al., 2007); however, they may be up-regulated or recruited during pathologic conditions or with specific visual or pharmacological stimuli (Jones et al., 2012; Sladek and Nawy, 2020; Xia et al., 2007). Our cobalt uptake experiments showed little signal in RGCs compared with AII-ACs. Moreover, transcript levels of *GRIA2* were markedly lower in AII-ACs compared with midget and parasol RGCs, and there was little difference in expression between On-GCs and their Off-type counterparts. Thus, the difference in the sensitivity of the On versus Off pathways to CP-AMPAR blockade cannot be explained by effects at the level of the cone bipolar cell to GC synapses.

If the critical CP-AMPARs are located on the AII-ACs, why doesn’t CP-AMPAR blockade affect Off-pathway signaling? AII-ACs make glycinergic connections with Off-CBCs as part of the primary rod pathway (Figure 1A) and thus one might expect that under background illumination, a sustained CP-AMPAR-mediated depolarization of AII-ACs would increase glycinergic inhibition onto Off-CBC terminals. Indeed, there is evidence that such glycinergic inhibition hyperpolarizes Off-CBCs below the threshold for glutamate release and thus contributes to the more rectified transmission of the Off-pathway (Liang and Freed, 2010; Zaghloul et al., 2003). Conversely, blocking CP-AMPARs might be expected to disinhibit the Off-CBCs allowing them to increase tonic glutamate release onto Off-type GCs, thereby increasing their background spiking rate (Wässle et al., 1986; Zaghloul et al., 2003). Such effects were not observed in the spiking responses of Off-GCs. In mouse and primate, certain Off-CBCs receive the majority of AII-AC output synapses, suggesting that not all Off-channels carry rod signals (Graydon et al., 2018; Jusuf et al., 2005; McLaughlin et al., 2021; Tsukamoto and Omi, 2015). Moreover, in mouse, AII-ACs dynamically filter signals from RBCs, such that transient and sustained release components are differentially relayed to downstream On- and Off-CBCs (Graydon et al., 2018). While transient and sustained components are faithfully transmitted to On-CBCs across a broad range of AII-AC membrane potentials, sustained signals are only relayed to Off-CBCs under specific network conditions when AII-ACs are relatively depolarized (Graydon et al., 2018). Another possible explanation for the lack of effect of CP-AMPAR block on the Off-GCs is that the primate Off-bipolar cell terminals are so rectified that the relief of glycinergic inhibition when blocking CP-AMPARs has little effect on tonic transmitter release. Further work will be required to test these hypotheses.

The observation that the CP-AMPAR antagonist blocked sustained firing in On-midget GCs during inhibitory blockade discounts the possibility that the effects of CP-AMPAR blockade result from indirect suppression of activity in other amacrine cell types. Moreover, although horizontal cells express CP-AMPARs (Osswald et al., 2007), direct blockade of photoreceptor to horizontal cell transmission should impact both the On- and Off pathways. Horizontal cells have also been reported to provide feedforward GABAergic inhibition that produces depolarizing responses in On-bipolar cells and hyperpolarizing responses in Off-bipolar cells due to differences in dendritic chloride gradients (Duebel et al., 2006). However, the effects of CP-AMPAR blockade persisted in the presence of GABAR blockers, and thus our results cannot readily be explained by effects on horizontal cells. Taken together, our findings are best explained by action of the CP-AMPAR antagonist at the rod bipolar to AII-AC synapse.

### Physiological significance of CP-AMPARs in the On-pathway

Blocking CP-AMPARs hyperpolarized On-GCs by blocking tonic excitation and there was a concomitant reduction in the variance of the EPSCs and EPSPs in the On-GCs. The reduced variance is consistent with the tonic excitation being generated by summation of many relatively large unitary EPSCs mediated by CP-AMPARs that have a relatively high single channel conductance (Swanson et al., 1997). Such a high variance might seem disadvantageous, as it would reduce the signal-to-noise ratio for graded changes in the membrane potential. However, at the On-CBC to On-GC synapse, a sustained signal in the On-CBC is converted into corresponding changes in the mean spike rate in the GC. The mean spike rate is presumably driven by the synaptic variance, which randomly produces EPSPs that exceed spike threshold in the GCs. As the GC depolarizes during a sustained light stimulus, many more EPSPs exceed the threshold and the spike rate increases. In this scenario, a high variance can be advantageous since the amplitude distribution of the EPSPs will be broad, and larger changes in the graded membrane potential will be required to produce a given change in firing rate. Thus, a high variance will allow for a larger dynamic range for signal transmission but with lower gain. Conversely, low variance will produce high gain but with correspondingly smaller dynamic range. Thus, the high variance is likely to be functionally important, but how the trade-off between dynamic range and gain is set by the demands of the system will be interesting to investigate in the future.

In addition to the tonic input mediated by CP-AMPARs, On-midget and On-parasol GCs displayed a major transient excitatory input that was resistant to the CP-AMPAR antagonist and gap-junction blockers (Figures 4 and 5). The EPSPs and EPSCs in the presence of the CP-AMPAR blocker lacked the high variance that is characteristic of the CP-AMPAR-sensitive component, with the result that the light responses were more rectified in favor of positive luminance changes. However, the peak of the transient excitatory conductance at the onset of the positive phase of the stimulus was comparable in control and during CP-AMPAR block (Figure 5C). With signals through the primary rod pathway suppressed, these excitatory inputs presumably arise from the direct cone pathway (cone→On-CBC→On-GC). While the sustained excitation seems well-suited to support relatively linear signaling at modest contrast fluctuations, the rectified synapses might be important to improve resolution of large positive contrasts. Both the On-midget and On-parasol GCs appear to show a similar arrangement, with sustained and transient excitatory inputs, suggesting that both GC types receive input from multiple bipolar cell types with diverse temporal properties (Tsukamoto and Omi, 2013).

In the primate retina, it has been reported that rod signals are routed to GCs via the primary rod pathway for backgrounds ranging from low scotopic to high mesopic levels (Grimes et al., 2018). This result is in contrast to other mammals where secondary (rod→cone gap junctions) and tertiary rod pathways (rod to Off-CBC) play a greater role under mesopic conditions (Grimes et al., 2018; Soucy et al., 1998; Tsukamoto et al., 2001). In contrast to the experiments described in Grimes et al. (2018), which used relatively short background adaptation times, our experiments were conducted with the retinal pigment epithelium (RPE) attached, making it possible to continuously expose the retina to the background light. Longer adaptation periods have been reported to reduce rod saturation and permit rod-driven GC responses even at high photopic backgrounds (Frederiksen et al., 2021; Pahlberg et al., 2017; Tikidji-Hamburyan et al., 2017). Thus, it will be important to determine whether CP-AMPARs play a role in On-GC signal linearity across a wider range of background levels. Given the conserved expression of CP-AMPARs in AII-ACs, it will also be interesting to examine the contribution of this synapse to On-GC signal linearity in other species.

### Limitations of the study

The data suggest that sustained signals, required to support linearity in the On-pathway, reach some On-GCs via the primary rod pathway, at least at relatively low light levels. At the light levels used, rod photoreceptors might be expected to contribute to signaling, but this leaves open the question as to what happens at higher light intensities where rods are expected to saturate but the On-pathway still displays linear signal transfer. An interesting and important direction for future studies will be to determine the range of light adaptation levels under which CP-AMPARs contribute to linearity of On-GC function.

## STAR★METHODS

### RESOURCE AVAILABILITY

#### Lead contact

Further information and requests for reagents and resources should be directed to and will be fulfilled by the lead contact, Teresa Puthussery (tputhussery@berkeley.edu).

#### Materials availability

This study did not generate new unique reagents.

#### Data and code availability

- All data reported in this paper will be shared by the lead contact upon request.
- This paper does not report original code.
- Any additional information required to reanalyze the data reported in this paper is available from the lead contact upon request.

### EXPERIMENTAL MODEL AND SUBJECT DETAILS

Adult female and male macaque (M. *mulatta*, *M*. *fascicularis*) eyes were obtained immediately *post-mortem* from the Oregon and California National Primate Research Center biospecimen distribution programs. Eyes from UC Berkeley were from animals that were euthanized for unrelated studies and were enucleated under terminal anesthesia in accordance with procedures approved by the Animal Care and Use Committee of the University of California, Berkeley and as specified in the National Research Council guidelines.

### METHOD DETAILS

#### Tissue preparation

The anterior eye and vitreous were removed immediately after enucleation and posterior eyecups were stored at room temperature in bicarbonate buffered Ames’ medium (US Biologicals) equilibrated with carbogen (95% O_2_/5% CO_2_) containing 1.5 mL of penicillin-streptomycin (10,000 units/mL Penicillin 10,000 μg/mL Streptomycin, Gibco). The retina, with attached pigment epithelium and choroid, was isolated from the sclera approximately 1 h later and stored at room temperature in bicarbonate buffered Ames’ until further use.

#### Retinal ganglion cell recordings and analysis

Pieces of retina with attached RPE and choroid (~10 × 8 mm; between 5 and 15 mm eccentricity) were placed on an Anodisc membrane ganglion cell side up (Anodisc 13 inorganic membrane disc, diameter 13 mm, pore size 0.2 μm, GE Whatman), transferred to the recording chamber and continually supplied with Ames’ medium (33–35°C) at a rate of ~4 mL/min. A tissue harp was placed on the retina to further stabilize it. To target cells for recording, preparations were illuminated with 700 nm or 870 nm light and visualized using gradient contrast optics. For extracellular loose-patch recordings, borosilicate glass microelectrodes (~5 MΩ resistance) were filled with Ames’ medium. For voltage-clamp recordings, electrodes were wrapped in Parafilm to reduce input capacitance and filled with a Cs+ based solution containing in mM: 117 Cs-methanesulfonate, 10 Na_0.5_-HEPES, 9 CsCl, 7 Na_2_-phosphocreatine, 3QX-314, 2 Mg-ATP, 1 Na-GTP, and 1 EGTA (adjusted to pH 7.35 with CsOH). Voltages were corrected for the liquid junction potential of −16 mV. In most recordings, 100 μM spermine was added to the intracellular solution, however, no obvious differences were observed in the linearity of excitatory currents between recordings made with and without spermine. Current clamp recordings were made using pipettes filled with a K+ based intracellular solution containing in mM: 118 K-methanesulphonate, 10 KCl, 10 Na_2_-phosphocreatine, 10 Na-HEPES, 2 Mg-ATP, 1 Na-GTP, and 1 EGTA (adjusted to pH 7.35 with KOH). Alexa-488 hydrazide (0.5 mM, Invitrogen) was added to the pipette solution to reveal cellular morphology of the GCs during some recordings. Current and voltage signals were filtered at a −3 dB cutoff frequency of 2.5 kHz by the 4 pole Bessel filter of an HEKA EPC-10 double patch amplifier and digitized at 10 kHz.

#### AII-amacrine cell recordings

For recordings from AII-ACs in retinal slices, dark-adapted retinal pieces (~5 × 5 mm) were separated from the choroid/RPE, mounted ganglion cell side down on nitrocellulose filter paper and sectioned at ~300 μm using a manual tissue chopper. Slices were oriented by stabilizing the filter paper in tracks of vacuum grease and transferred to the recording chamber where they were continuously superfused with Ames’ medium warmed to 32–33°C. Slices were visualized with 870 nm infrared illumination with Dodt gradient contrast optics on an Olympus BX-51 WI microscope. Borosilicate pipettes were pulled to 9–12 MΩ and filled with a cesium based intracellular solution, containing (in mM): 114 CsMeSO4, 10 Na_0.5_-HEPES, 1 EGTA, 9 CsCl, 2 Mg-ATP, 0.3 Na-GTP, 10 phosphocreatine, and 0.1 Alexa 488 hydrazide (pH to 7.35 with CsOH). AII-ACs were targeted based on their soma position (adjacent to IPL), larger soma size, and presence of a prominent proximal dendrite. AII-ACs could be distinguished upon establishing the whole-cell recording configuration by: 1) the presence of unclamped action-currents in response to a +5 mV test pulse consistent with the presence of voltage-gated sodium channels (Boos et al., 1993; Veruki and Hartveit, 2002; Tian et al., 2010; Wu et al., 2011) and their high frequency of large amplitude spontaneous EPSCs (Veruki et al., 2003). Cell morphology was confirmed at the end of recordings. For agonist application, 0.5 mM L-Glu was applied to stratum 5 of the IPL using brief (20 ms) pressure pulses (Picospritzer III, Parker Hannifin) from a ~10 MΩ pipette. Currents were filtered at a −3 dB cut-off frequency of 2 kHz by the 4 pole Bessel filter of a HEKA EPC-10 double patch amplifier. Series resistance was uncompensated but cells were excluded from analysis if series resistance exceeded 35 MU. A liquid junction potential correction of −15 mV was applied for all analyses.

#### Pharmacology

For pharmacological experiments, concentrated drug stocks were prepared ahead of time and stored at −20°C until use. Measurements of drug effects were made at least 3 min after wash-in to ensure complete bath equilibration.

#### Light stimulation

Prior to recording light-evoked responses, the microscope condenser illumination was switched off. Light stimuli, generated on a monochromatic OLED microdisplay (Emagin; peak λ = 518 nm), were projected onto the preparation through the microscope objective (Olympus water immersion, 10x/0.30 N.A.) The maximum intensity of the OLED display was approximately 1,200 photons/μm^2^/s on the retina after attenuation through a 2 log-unit neutral density filter. Light output was linearized using a calibrated look-up table. The background light on the stimulus display was always present and was set to half the maximal intensity (~600 Rh*/rod/s, assuming a collecting area of 1μm^2^ for rods [Schneeweis and Schnapf, 1995]). Stimuli were modulated at 80% contrast, where percentage contrast was defined as 100 × (L_max_ − L_min_)/L_background_, where L_max_ and L_min_ are the maximum and minimum intensities of the stimulus. Stimuli were square-wave contrast-modulated (1Hz), centered spots of light with a diameter that corresponded to the size of the excitatory receptive field center. Receptive field center-size was estimated from area-response functions constructed from spike responses.

#### Cobalt assay and immunohistochemistry

The cobalt assay procedure was performed as described previously with minor modifications (Aurousseau et al., 2012). All steps were performed at 22°C in solutions equilibrated with 95% O2 / 5% carbon dioxide buffer. Pieces of macaque peripheral retina (~4 × 4 mm), with choroid and RPE attached, were dissected in Ames’ medium then transferred to a carbogenated assay buffer containing (in mM): 57.5 NaCl, 5 KCl, 20 NaHCO_3_, 12 D(+) glucose, 139 sucrose, 0.75 CaCl_2_, and 2 MgCl_2_ (pH 7.4) and incubated for 60 min at 22°C. Samples were then incubated in either: 1) assay buffer only or 2) assay buffer containing 80 μM GYKI 53655 for 30 min. For cobalt loading, 10 mM L-glutamate and 5 mM CoCl_2_ were added to all samples for 15 min. Following loading, excess cobalt was chelated with 2 mM EDTA for 5 min, samples were washed in assay buffer and cobalt ions were precipitated with 0.24% (NH4)_2_S for 5 min. After final washes in assay buffer, retinas were fixed in 4% paraformaldehyde in 0.1 M phosphate buffer for 120 min at 22°C. After fixation, samples were washed in phosphate buffered saline, cryoprotected in graded sucrose solutions (10%, 20%, and 30%), embedded and frozen in Cryogel medium (Leica), sectioned at 10–16 μm and stored at −20°C until further use. For cobalt detection, the cobalt reaction product was silver intensified (#SE100, Sigma-Aldrich) for ~35–40 min then fixed with Na_2_S_2_O_3_ for 2 min. Cobalt staining patterns were assessed in 15 sections from different regions of a single retina. Immunohistochemistry was performed after silver-intensification of the cobalt signal. Briefly, sections were washed with PBS, blocked for 1 h in a blocking buffer containing 10% normal donkey serum, 1% Tx100, 0.025% NaN_3_ in PBS, then incubated overnight at 22°C in primary antibodies diluted in 3% normal donkey serum, 1% Tx100, 0.025% NaN_3_ in PBS. After washing, secondary antibodies, raised in donkey and conjugated to Alexa Fluor 488 or 594 were applied for 1 h at 22°C. After final washes, samples were incubated in Hoescht 33342 for 5 min before coverslipping in Mowiol.

#### Fixed tissue microscopy

Retinas containing ganglion cells filled during recordings were fixed in 4% paraformaldehyde for 30 min at room temperature, mounted in Mowiol and imaged on an Olympus Fluoview 1000 confocal microscope with a UPLFLN 40x oil/N.A.1.3 objective with the 488 laser line. Immunolabeled retinal sections from cobalt assay experiments were imaged on a Zeiss LSM 880 laser scanning confocal microscope with a Zeiss Plan-Apochromat 20x/0.8 objective using the 405, 488 and 591nm laser lines for excitation. Transmitted images were collected with a substage transmitted detector fitted with Dodt contrast optics. Z-stacks were collected with a z-voxel size of 1.4 μm and z-interval of 0.68 μm. Fluorescence images are maximum projections of 4 sections. Linear adjustments to brightness and contrast were made in FIJI and figure layouts were composed with the EzFig plugin for FIJI.

#### Transcriptomic analysis

Visualizations of single-cell RNA-sequencing expression profiles from peripheral primate retinal ganglion cells and amacrine cells were generated from a published dataset (Peng et al., 2019) using the Single Cell Portal (Broad Institute). For transcriptomic data, violin/box plots were generated in Igor Pro.

### QUANTIFICATION AND STATISTICAL ANALYSIS

#### Spike counts

Spikes were detected by taking the time-derivative of the extracellular voltage recordings to remove slow baseline drift. We then calculated the standard deviation of each trace and set a threshold at 3 SD to detect spike times. For measurements of the SD of voltage signals in Figure 4, spikes were “blanked” to reduce their contribution to the measurement. Blanking was accomplished by linear interpolation from the data point 1 ms preceding the spike-time until 3 ms afterwards. This process removed the large depolarizing transient but did not entirely remove the slow depolarization leading to each spike, or the after-hyperpolarization following each spike. Thus, the SD measurements shown in Figure 4 don’t simply represent the sub-threshold voltage noise, but still reflect in part any change in spike-rate. Peristimulus spike time histograms (PSTHs) were produced from multiples of 20 stimulus trials for a given experimental condition using a bin width of 20 ms. Membrane current-variance estimates were made from current records at the liquid junction potential corrected holding potential of −76 mV.

#### Conductance analysis

Synaptic conductances were estimated as described previously (Buldyrev et al., 2012; Manookin et al., 2010; Taylor and Vaney, 2002; Venkataramani et al., 2014). Briefly, current-voltage (I-V) relations of the net light-evoked synaptic currents were measured at 10 ms intervals from responses recorded at a range of membrane potentials from −110 to +50 mV. Synaptic conductances were estimated from fits to the I-V relations, assuming a reversal potential for excitation of 0 mV and inhibition of −70 mV. Membrane potentials were corrected for a liquid junction potential of −13 mV. The parameters used to account for the non-linear I-V relation of the NMDAR-mediated synaptic currents were taken from a previous publication (Buldyrev et al., 2012).

#### Statistical analysis

For statistical analysis of electrophysiological data, data distributions were tested for normality using the Shapiro-Wilks test and control and drug conditions were compared using paired t-tests except where otherwise noted. An alpha level of 0.05 was applied for all statistical comparisons. For transcriptomic data, comparisons were made using student’s unpaired *t-test* (Mou et al., 2019) with Bonferroni correction for multiple comparisons. Exact p-values are noted in the results text and p-values in the figures are denoted with asterisks as follows: *<0.05, **<0.01, ***<0.001. All analysis and statistical tests were performed using Igor Pro 9.0 (Wavemetrics). Measurements are listed as the mean ± the standard deviation (s.d.). Error bars and shaded areas on peristimulus spike time histograms show ± 1 s.d. of the mean. n values refer to number of cells.

## Figures and Tables

**Table:** KEY RESOURCES TABLE

REAGENT or RESOURCE	SOURCE	IDENTIFIER
Antibodies		
Goat anti-calretinin	Chemicon/Millipore	Cat# AB1550 RRID:AB_90764
Guinea pig anti-RBPMS	Phosphosolutions	Cat# 1832-RBPMS RRID:AB_2492226
Donkey anti-guinea pig Alexa Fluor 594	Molecular Probes/ThermoFisher	Cat# A-11055 RRID:AB_2534102
Donkey anti-goat Alexa Fluor 488	Jackson ImmunoResearch Labs	Cat# 706-585-148 RRID:AB_2340474
Biological samples		
Macaque retina (*M. mulatta, M. fascicularis*)	Oregon National Primate Research Center	N/A
Macaque retina (*M. mulatta, M. fascicularis*)	California National Primate Research Center	N/A
Macaque retina (*M. mulatta*)	University of California, Berkeley	N/A
Chemicals, peptides, and recombinant proteins		
Ames’ medium with L-glutamine	US Biologicals	Cat# A1372
Alexa 488 hydrazide	Invitrogen	Cat# A10436
IEM 1460	Tocris Bioscience	Cat# 1636
D-AP5	Abcam Cat# ab120003 Tocris Bioscience	Cat# 0106
L-glutamic acid (L-glutamate)	Sigma-Aldrich	Cat# G8415
Ammonium sulfide (NH4)_2_S solution	Sigma-Aldrich	Cat# 515809–100ML
SR95531 (6-Imino-3-(4-methoxyphenyl)-1(6H)-pyridazinebutanoic acid)	Abcam	Cat# ab120042
TPMPA (1,2,5,6-Tetrahydropyridin-4-yl) methylphosphinic acid)	Tocris Bioscience	Cat# 1040
Strychnine	Sigma-Aldrich	Cat# S0532
GYKI 53655	Abcam	Cat# 120490
Silver intensification kit	Sigma-Aldrich	Cat# SE100
Software and algorithms		
Igor Pro 9.0	Wavemetrics	https://www.wavemetrics.com/
Adobe Illustrator	Adobe	N/A
HEKA Patchmaster	HEKA	https://www.heka.com/downloads/downloads_main.html#down_patchmaster
ImageJ (FIJI)		https://imagej.nih.gov/ij/ RRID:SCR_003070
Single Cell Portal	Broad Institute	https://singlecell.broadinstitute.org/single_cell
EzFig Plugin for ImageJ (v1.2)	Benoit Aigouy	https://github.com/baigouy/EZFig

## Highlights

- AII amacrine cells express calcium-permeable AMPA receptors (CP-AMPARs)
- Blocking CP-AMPARs suppresses sustained, light-driven signals in On-ganglion cells
- CP-AMPARs on AII-ACs may support the linear signaling specific to On-ganglion cells

## INCLUSION AND DIVERSITY

We support inclusive, diverse, and equitable conduct of research.
